# A favorable impression of vaccination leads to a better vaccination rate for the human papillomavirus vaccine: A Japanese questionnaire survey investigation

**DOI:** 10.1016/j.jvacx.2022.100254

**Published:** 2022-12-23

**Authors:** Sinchul Jwa, Yoshihiko Yuyama, Hisako Yoshida, Takashi Hamazaki

**Affiliations:** a Department of Pediatrics, Osaka Metropolitan University Graduate School of Medicine, 1-4-3 Asahi-machi, Abeno-ku, Osaka-shi, Osaka 545-8585, Japan; b Department of Medical Statistics, Osaka Metropolitan University Graduate School of Medicine, 1-4-3 Asahi-machi, Abeno-ku, Osaka-shi, Osaka 545-8585, Japan

**Keywords:** Childhood vaccines, HPV vaccine, Vaccine knowledge, Vaccine hesitancy, ACME, average causal mediation effects, ADE, average direct effects

## Abstract

**Background:**

The Japanese vaccination rate for infants and children is seemingly excellent, except for the human papillomavirus (HPV) vaccine. Regardless of its efficacy, the inoculation rate in Japan has dropped to approximately 1 % since 2013 because of negative information about vaccine side effects. We aimed to demonstrate the factors that lead to low vaccine acceptance rates (e.g., caregiver attitudes, popular misconceptions) to inform the relevant target demographic of a stronger intention to immunize and to facilitate recovery of HPV vaccine coverage.

**Methods:**

We conducted this study using data from two questionnaire surveys. Statistical analyses of factors affecting vaccine impressions and mediation effects of HPV vaccine impressions were performed. The difference in the knowledge about each vaccine was evaluated.

**Results:**

Of the respondents, 95.9 % reported their intent to vaccinate their infants, whereas 58.2–78.3 % felt that they sufficiently understood the aims, efficacy, and risks of vaccination and 66.6 % had a positive impression of vaccines. Overall, 21.3 % of parents responded that they planned to have their child vaccinated against HPV, and 25.8 % had a favorable impression of this vaccine. Among factors affecting vaccine impressions, we found that parents had anxiety about vaccines when they felt that their knowledge of vaccines was insufficient. Additionally, impressions of the HPV vaccine had a mediating effect on the association between the impressions of infant vaccines and parents’ intent to provide the vaccine to their children.

**Conclusion:**

These findings show that as a society, we need to improve impressions and knowledge regarding vaccines, including but not exclusively the HPV vaccine. Moreover, although the recovery of HPV vaccine coverage is strongly desired for improving public health, simply improving impressions about the HPV vaccine or educating parents with substantive knowledge is insufficient. Instead, improving impressions and understanding of the vaccination itself is necessary.

## Introduction

Vaccination is crucial for public health. Through vaccination, we can now prevent many infections and their complications that adversely affect children’s health [1]. In Japan, the national immunization program offers both routine and voluntary vaccines. Currently, children are administered a total of ten routine vaccines, including the human papillomavirus (HPV) vaccine, with financial support from the government; voluntary vaccines without compensation are also available. Almost all vaccines are administered before the child’s third birthday.

Cancer can be prevented through immunization. As cancer is the leading cause of mortality in Japan and the second leading cause of mortality worldwide, vaccine-based cancer prevention programs have extraordinary relevance. In particular, the HPV vaccine prevents more than 90 % of all infections arising from oncogenic virus types, which effectively leads to cancer prevention, and other immunotherapy-based vaccinations are under development as well [2], [3], [4], [5].

However, regardless of its effectiveness, some people evade vaccination due to various unfounded concerns. Moreover, although the inoculation rate for almost all routine vaccines is approximately 95 %, voluntary vaccines (e.g., mumps vaccine) have inoculation rates of approximately 40–50 % [6], [7], [8]. In the case of the HPV vaccine, although it was introduced as a routine vaccine in Japan in 2013, due to misinformation in the mass media, the coverage has dropped to approximately 1 % since 2013 [9], [10]. In media reports, some events have been improperly associated with vaccination.

One of the biggest causes of suboptimal vaccine coverages is vaccine hesitancy, which is defined as “the delay in acceptance or refusal of vaccines despite the availability of vaccine services”[11], [12]. In addition, vaccine hesitancy is a broader concept, including parents’ concerns or negative attitudes on vaccination even if their children are vaccinated[13], [14]. Gaining public trust is required to address and reduce vaccine hesitancy to improve vaccination rates, and thus, better communication of scientific information is vital[15], [16], [17], [18], [19], [20], [21], [22], [23]. Although various approaches, such as administering educational information sheets at well-child visits [24], [25], [26], have been proposed for improving the vaccination rate, there has been no meaningful impact on the situation as a result of these interventions. However, it is integral to improve inoculation rates for the reasons mentioned above. Therefore, we conducted this study to elucidate the misconceptions and attitudinal/educational factors that hinder vaccination, specifically the HPV vaccine, in routine vaccine programs.

The primary aim of the present study was to conduct an exploratory analysis to comprehensively evaluate the concerns surrounding vaccination, thereby establishing better public trust regarding vaccination and enabling the effective implementation of routine vaccination programs. The secondary aim of this study was to identify factors related to low HPV vaccine uptake rates and provide valuable information that may be relevant to the recovery of HPV vaccine coverage in Japan. In this study, we performed statistical analyses to determine the relationships among parents’ characteristics, knowledge, vaccine impressions, and the willingness to provide the HPV vaccine to children. Furthermore, we analyzed the relationship between the impression of childhood vaccines and the intent to provide the HPV vaccine to children.

## Materials and methods

### Study source

We utilized data from two questionnaire surveys conducted by Ninps Lab, an information and educational organization in Japan, through a website currently managed by SOLVE Media Inc. (Tokyo, Japan). Hence, there was no preliminary study to prepare a questionnaire or decide the sample size. The participants were parents of children younger than 3 years or expectant parents who registered for membership at Ninps Lab and voluntarily answered questions on which vaccines their youngest child would receive. In the second survey, Ninps Lab separately contacted the respondents of the first questionnaire survey; these participants were invited to participate in the second round of the study even if their children were older than 3 years. For answering the questions, participants could not move on to the next question without responding to the previous one.

Notably, during this study, Ninps Lab was managed by Polar Star K.K. (Tokyo, Japan), which also manages Mil Care Corporation under the supervision of medical doctors and has already conducted collaborative research with several universities as well as the Japan Broadcasting Corporation (Tokyo, Japan).

### Questionnaire details

The questionnaire included the following questions: participants’ information; their impression of each vaccine; the extent to which participants understood the efficacy, the value, and the risk associated with each vaccine; the willingness of the participants to know the details of each vaccine; the vaccination status of kids or parents’ intent to vaccinate, and the information source of vaccines.

### Participants

The first survey was administered between July 30th to August 27th, 2019, and the second round of the study was conducted from October 1st to 20th in 2020. Every parent voluntarily answered an online questionnaire with questions on their children’s projected vaccine schedules for 2 years comprising the survey periods. The questionnaires collected information on participants' background, impressions of vaccines, and relevant information sources. In the second questionnaire, participants answered questions about the HPV vaccine specifically in the following section on childhood vaccination in addition to the questions in the first questionnaire on HPV vaccine impressions, intentions to vaccinate kids against HPV in the future, and participants’ information sources about the HPV vaccine. Five participants of all the respondents of each survey were compensated with a gift card of 500 JPY by lottery.

### Ethics statement

All participants were informed after the questionnaire surveys that anonymized and deidentified answers would be utilized for analyses and that they could freely opt out of our study. The survey responses were anonymized and deidentified when the data were delivered to us (i.e., only Ninps Lab had access to participant identifiers). This study was approved by the Institutional Review Board of the Osaka City University Graduate School of Medicine (approval number: 2020–161) and was conducted in accordance with the principles of the Declaration of Helsinki and its later amendments.

### Data analysis

All statistical analyses were performed using R version 4.0.3 (The R Project for Statistical Computing, Vienna, Austria). We conducted linear regression analysis and considered differences to be statistically significant if two-sided *p*-values were<0.05.

We confirmed mediation effects using the “mediation” package in R [27]. Additionally, we examined differences in individual understanding of each vaccine with regard to a reference vaccine (*H. influenzae* type b [Hib]) using a generalized least squares model with the assumption of a compound symmetrical correlation structure. We calculated Cronbach’s alpha to validate the questionnaire’s reliability.

We used variables related to vaccine impression as explanatory variables, the score of willingness to know about vaccines and knowledge of each vaccine, and information sources of vaccines for assessment. To analyze the impression of the HPV vaccine, we used childhood vaccine impressions, the score for willingness to learn about the HPV vaccine, knowledge of the HPV vaccine, and information sources of the HPV vaccine. The HPV vaccine impression was an explanatory variable to assess parents’ intention to administer it to their kids.

## Results

### Participant characteristics

**Fig. 1:**
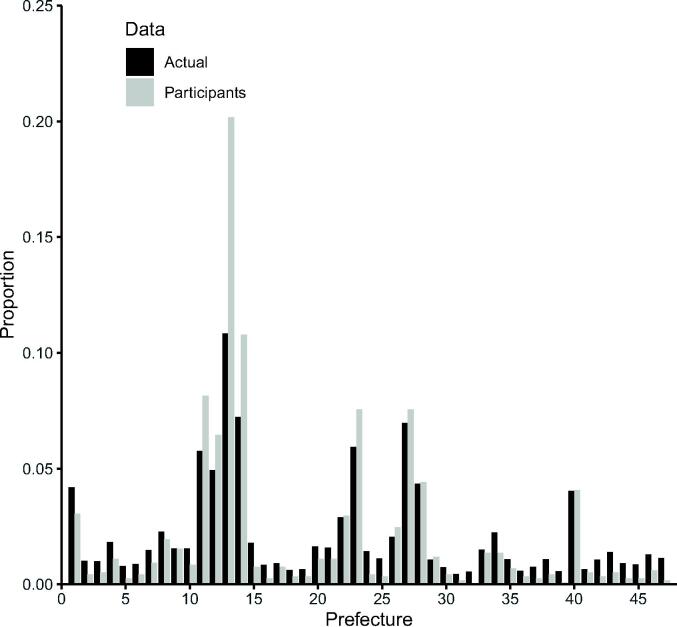
**The actual and participants population in each prefecture in Japan. Prefecture:** 1 Hokkaido; 2 Aomori; 3 Iwate; 4 Miyagi; 5 Akita; 6 Yamagata; 7 Hukushima; 8 Ibaraki; 9 Tochigi; 10 Gunma; 11 Saitama; 12 Chiba; 13 Tokyo; 14 Kanagawa; 15 Niigata; 16 Toyama; 17 Ishikawa; 18 Fukui; 19 Yamanashi; 20 Nagano; 21 Gifu; 22 Shizuoka; 23 Aichi; 24 Mie; 25 Shiga; 26 Kyoto; 27 Osaka; 28 Hyogo; 29 Nara; 30 Wakayama; 31 Tottori; 32 Shimane; 33 Okayama; 34 Hiroshima; 35 Yamaguchi; 36 Tokushima; 37 Kagawa; 38 Ehime; 39 Kouchi; 40 Fukuoka; 41 Saga; 42 Nagasaki; 43 Kumamoto; 44 Oita; 45 Miyazaki; 46 Kagoshima, and 47 Okinawa.

The numbers of answers for the first and the second surveys were 565 and 629, respectively. A total of 67 participants responded to both surveys. As these 67 participants answered the questions in the first survey, their responses were included twice, and we used the latest data for these overlapping questions herein. Additionally, after excluding duplicated answers, 497 and 681 answers in the first and second surveys, respectively, were analyzed. The proportion of the population in each prefecture where participants lived was similar to the actual number, however, slightly skewed in the metropolitan area; there was no respondent in the Shimane prefecture (Fig. 1; No.11-14). Every participant answered all survey questions, and there was no missing case. The participants’ characteristics, general impressions, willingness to provide the vaccine, and information sources regarding vaccines are summarized in Table 1. The respondents’ knowledge and willingness to provide each vaccine are presented in Table 2. At the time that this research was conducted, the rotavirus, mumps virus, and flu virus vaccines were voluntary vaccines, whereas others were routine vaccines.

**Table 1:** Answers to the questionnaire questions regarding general topics.

**Age**				
	32 (29–35)	1178		
**Which sibling is the kid whom you are answering about?**		**N (%)**		
	1	841 (71.4)		
	2	263 (22.3)		
	3	66 (5.6)		
	greater than4	8 (0.7)		
**Have you administered, or are you going to administer, each vaccine that is for children aged 0**–**2 years to your kids?**				
	All vaccines, including voluntary vaccines	921 (78.2)		
	Only routine vaccines	209(17.7)		
	Will decide whether a vaccine will be provided one by one	28 (2.4)		
	Not willingly	8 (0.7)		
	Generally will not provide vaccines	2 (0.2)		
	Have not decided yet	10 (0.8)		
**Do you know that the HPV vaccine is one of the routine vaccines?**				
1	Exactly	246 (36.5)		
	Somehow	181 (26.6)		
2	Neither	20 (2.9)		
3	Not very much	136 (20.0)		
	No	95 (14.0)		
**What is your information source for each vaccine? (choose 3 options)**		**Childhood Vaccines**	**HPV Vaccine**	
Official sources	Doctor	915 (77.7)	485 (71.2)	
	Notice from the local government	642 (54.5)	297 (43.6)	
	Maternal and Child Health Handbook	638 (54.2)	158 (23.2)	
Unofficial online sources	Internet site	374 (31.7)	345 (50.7)	
	Social networking service	73 (6.2)	69 (10.1)	
Neighbors	Friends	181 (15.4)	90 (13.2)	
	Family	123 (10.4)	41 (6.0)	
Media	Parenting books	125 (10.6)	89 (13.1)	
	Mass media	3 (0.3)	30 (4.4)	
	Experts	17 (1.4)	28 (4.1)	
**What is your impression about each vaccine?**		**Childhood Vaccines**	**HPV Vaccine**	
1	Very positive	324 (27.5)	50 (7.3)	
	Positive	461 (39.1)	125 (18.4)	
2	Neutral	239 (20.3)	184 (27.0)	
			67 (9.8)	
3	Negative	140 (11.9)	198 (29.1)	
	Very negative	14 (1.2)	57 (8.4)	
**Do you wish for detailed information about each vaccine?**		**Routine Vaccines**	**Voluntary Vaccines**	**HPV Vaccine**
1	As much as possible	745 (63.2)	803 (68.2)	446 (65.5)
	Rather yes	371 (31.5)	325 (27.6)	194 (28.5)
2	Neutral	54 (4.6)	42 (3.5)	30 (4.4)
3	Rather no	8 (0.7)	7 (0.6)	7 (1.0)
	Not at all	0 (0.0)	1 (0.1)	4 (0.6)

**Abbreviation:** HPV, human papillomavirus.

**Table 2:** Questions about each vaccine.

**Have you given, or are you going to give, each vaccine to your children?**			**How much do you understand about the efficacy,** **value, and risk of each vaccine?**		
Hib					
1	Yes	1105 (93.8)	1	Very Well	101 (8.6)
				Well	584 (49.6)
2	Have not decided yet/do not remember	10 (0.9)	2	neither	164 (13.9)
3	No	63 (5.3)	3	Poor	196 (16.6)
				Very Poor	133 (11.3)
PCV					
1	Yes	1111 (94.3)	1	Very Well	105 (8.9)
				Well	615 (52.2)
2	Have not decided yet/do not remember	10 (0.9)	2	neither	169 (14.3)
3	No	57 (4.8)	3	Poor	173 (14.7)
				Very Poor	116 (9.9)
HBV					
1	Yes	1115 (94.6)	1	Very Well	118 (10.0)
				Well	655 (55.6)
2	Have not decided yet/do not remember	10 (0.9)	2	neither	172 (14.6)
3	No	53 (4.5)	3	Poor	151 (12.8)
				Very Poor	82 (7.0)
DPT-IPV					
1	Yes	1113 (94.5)	1	Very Well	105 (8.9)
				Well	598 (50.8)
2	Have not decided yet/do not remember	11 (0.9)	2	neither	181 (15.4)
3	No	54 (4.6)	3	Poor	191 (16.2)
				Very Poor	103 (8.7)
BCG					
1	Yes	1108 (94.1)	1	Very Well	130 (11.0)
				Well	630 (53.5)
2	Have not decided yet/do not remember	15 (1.3)	2	neither	161 (13.7)
3	No	56 (4.6)	3	Poor	171 (14.5)
				Very Poor	86 (7.3)
MR					
1	Yes	1086 (92.2)	1	Very Well	171 (14.5)
				Well	661 (56.1)
2	Have not decided yet/do not remember	20 (1.7)	2	neither	163 (13.8)
3	No	72 (6.1)	3	Poor	120 (10.2)
				Very Poor	63 (5.4)
Varicella					
1	Yes	1069 (90.7)	1	Very Well	167 (14.2)
				Well	661 (56.1)
2	Have not decided yet/do not remember	28 (2.4)	2	neither	167 (14.2)
3	No	81 (6.9)	3	Poor	124 (10.5)
				Very Poor	59 (5.0)
Rotavirus					
1	Yes	942 (79.9)	1	Very Well	205 (17.4)
				Well	602 (51.1)
2	Have not decided yet/do not remember	108 (9.2)	2	neither	137 (11.6)
3	No	128 (10.9)	3	Poor	138 (11.7)
				Very Poor	96 (8.2)
HPV					
1	Yes	145 (21.3)	1	Very Well	28 (4.1)
				Well	214 (31.4)
2	Have not decided yet	364 (53.4)	2	neither	153 (22.5)
3	No	172 (25.3)	3	Poor	188 (27.6)
				Very Poor	98 (14.4)
Mumps virus					
1	Yes	913 (77.5)	1	Very Well	174 (14.8)
				Well	618 (52.5)
2	Have not decided yet/do not remember	84 (7.1)	2	neither	184 (15.6)
3	No	181 (15.4)	3	Poor	142 (12.0)
				Very Poor	60 (5.1)
Flu					
1	Yes	756 (64.2)	1	Very Well	282 (24.0)
				Well	640 (54.3)
2	Have not decided yet/do not remember	160 (13.6)	2	neither	142 (12.0)
3	No	262 (22.2)	3	Poor	78 (6.6)
				Very Poor	36 (3.1)

**Abbreviations:** Hib, *Haemophilus influenzae* type b; PCV, pneumococcal conjugate vaccine; HBV, hepatitis B virus; DPT-IPV, diphtheria/pertussis/tetanus/inactivated polio vaccine; BCG, Bacille de Calmette et Guérin; MR, measles/rubella; HPV, human papillomavirus.

Overall, 95.9 % of the survey respondents answered that they would provide all standard vaccines to their children through 3 years of age. However, only 66.6 % of parents had a positive impression of vaccination. Additionally, although 90.7–94.6 % of respondents planned to provide each routine vaccine for their child, only 58.2–70.6 % of respondents answered that they understood their risks and efficacies (Table 2). For each voluntary vaccine, the percentages of parents who were planning to get their children vaccinated and understood the corresponding risks and efficacies were 64.2–79.9 % and 67.3–78.3 %, respectively. Moreover, only 21.3 % of parents responded that their children would be provided the HPV vaccine, and 25.8 % of the questionnaire respondents had a positive impression of the vaccine; notably, these values were much higher than the actual vaccine inoculation rate for HPV in Japan.

As to the information sources, parents generally chose official sources as their primary source of childhood vaccine information, however, for the HPV vaccine, parents chose unofficial online sources on the HPV vaccine as their primary information source rather than the official handbook. Next, we statistically evaluated factors affecting the parents’ impressions of childhood vaccines, inparticular the HPV vaccine. In addition, we examined factors affecting the parents’ decision-making as to whether their children would receive the HPV vaccine.

### Questionnaire reliability and linear regression analysis

The reliability of these questionnaire surveys was tested using Cronbach’s alpha via the R “psych” package. The Cronbach’s alpha values for the 2019 survey and 2020 survey were 0.91 and 0.86, respectively. We thus confirmed the reliability of these questionnaires.

We conducted a linear regression analysis to examine factors that could affect the parents’ impressions of vaccines. We assigned points for each answer on the following scale: 1, positive; 2, neutral; and 3, negative (Table 1, Table 2). We evaluated the associated mean values in analyzing impressions. Table 3 presents the results of the linear regression analysis for the questionnaire and summarizes the information on parents answering about their first child. The results of the linear regression analysis indicated that the parents’ impressions of vaccination became worse when they thought that their knowledge of vaccines (especially routine vaccines) was insufficient or when they reported obtaining information about vaccines from neighbors, family, or friends (*p* = 0.006 and *p* < 0.001, respectively). In contrast, for parents who had more than one child, their impression of vaccines was worse when they received information about vaccines from their neighbors (*p* < 0.001).

**Table 3:** Linear regression analysis of the questionnaire.

**Childhood Vaccine Impression**				
	**Multiparas**		**Primiparas**	
	Coefficient (95 % CI)	*p*-value	Coefficient (95 % CI)	*p*-value
Willingness to know about routine vaccines	0.02 (−0.25, 0.29)	0.893	−0.17 (−0.42, 0.09)	0.197
Willingness to know about voluntary vaccines	0.03 (−0.29, 0.34)	0.866	−0.09 (−0.34, 0.17)	0.512
Mean score of the knowledge of routine vaccines	0.07 (−0.11, 0.25)	0.436	0.15 (0.04, 0.26)	0.006
Mean score of the knowledge of voluntary vaccines	0.12 (−0.08, 0.32)	0.229	0.11 (−0.01, 0.23)	0.069
Age	0.00 (−0.02, 0.02)	0.910	0 (−0.01, 0.01)	0.635
**Information source**				
Expert	−0.25 (−0.7, 0.19)	0.268	0.51 (0.02, 0.99)	0.041
Official source	−0.25 (−0.66, 0.16)	0.233	0.18 (−0.09, 0.45)	0.190
Unofficial online sources	0.08 (−0.08, 0.24)	0.354	0.03 (−0.07, 0.13)	0.547
Neighbors	0.42 (0.24, 0.6)	<0.001	0.18 (0.07, 0.29)	<0.001
Books or mass media	−0.13 (−0.39, 0.13)	0.320	0.07 (−0.08, 0.22)	0.348
**About HPV Vaccine**				
	**The impression**		**The willingness**	
	Coefficient (95 % CI)	*p*-value	Coefficient (95 % CI)	*p*-value
The impressions of childhood vaccines	0.25 (0.15, 0.34)	<0.001	0.03 (−0.05, 0.1)	0.450
The impressions of the HPV vaccine			0.35 (0.29, 0.41)	<0.001
The willingness to know about the HPV vaccine	0.17 (−0.01, 0.35)	0.060	0.24 (0.09, 0.38)	0.001
Know that the HPV vaccine is one of the routine vaccines	−0.01 (−0.08, 0.07)	0.847	0.08 (0.02, 0.14)	0.010
Know about the risk and efficacy of the HPV vaccine	0.03 (−0.05, 0.1)	0.489	−0.07 (−0.13, 0)	0.035
Age	0.02 (0, 0.03)	0.014	0.01 (0, 0.02)	0.071
Exdelivery	0.18 (0.05, 0.3)	0.006	−0.08 (−0.18, 0.02)	0.135
**Information source**				
Expert	0.38 (0.09, 0.67)	0.009	−0.04 (−0.27, 0.19)	0.738
Official source	0.1 (−0.05, 0.26)	0.188	−0.19 (−0.32, −0.07)	0.002
Unofficial online sources	0.06 (−0.05, 0.18)	0.292	0.01 (−0.09, 0.1)	0.881
Neighbors	0.09 (−0.06, 0.24)	0.234	0.03 (−0.09, 0.15)	0.615
Books or mass media	0.22 (0.06, 0.37)	0.005	0.02 (−0.1, 0.15)	0.722

**Abbreviations:** HPV, human papillomavirus; CI, confidence interval.

Next, we examined factors affecting the parents’ decisions to provide the HPV vaccine to their children. The results of linear regression analysis indicated that parents were apt to want their children to be vaccinated against HPV when they had a better impression of the HPV vaccine (*p* < 0.001), were willing to receive more information about the vaccine (*p* = 0.001), knew that the vaccine was one of the routine vaccines (*p* = 0.011), and/or utilized information from official information sources, such as doctors, the local government, or the Maternal and Child Health Handbook (*p* = 0.002). In contrast, when parents answered that they understood the risk, efficacy, and purpose of the HPV vaccine, they tended to have a negative attitude toward having their children vaccinated (*p* = 0.028).

Moreover, we confirmed the mediation effects for impressions of immunization (Table 4). This analysis revealed that impressions about the HPV vaccine had a mediation effect between impressions regarding standard immunization programs for infants and parents’ intent to provide an HPV vaccine for their children in the future, about whom they had answered in the questionnaire (*p* < 0.001); there was no direct effect of vaccine impressions for routine infant vaccines and the decision as to whether or not the HPV vaccine should be provided to children in adolescence. Overall, a good impression of immunization led to a positive intent towards the provision of the HPV vaccine specifically. This result implies that, to achieve better HPV vaccine coverage, it is necessary to improve impressions regarding the vaccination itself (rather than improving impressions about one particular vaccine).

**Table 4:** Mediation analysis (nonparametric bootstrap).

	Estimate	*p*-value
ACME	0.086 (0.054, 0.12)	<0.001
ADE	0.029 (−0.0450, 0.10)	0.448
Total Effect	0.11 (0.037, 0.19)	0.006
Prop. Mediated	0.75 (0.41, 2.08)	0.006

Treat: vaccine impressions; mediator: HPV vaccine impressions; outcome: willingness to provide the HPV vaccine; simulation: 10,000.

**Abbreviations:** ACME, average causal mediation effects; ADE, average direct effects; CI, confidence interval.

## Discussion

In this study, we examined vaccination status and vaccine impressions/misconceptions via questionnaire surveys administered to over 1,200 parents in Japan. To our knowledge, few surveys have examined parents’ attitudes toward routine vaccination in Japan that have been conducted on a population scale. We conducted this survey to further the understanding of the current situation in Japan, to better understand the current attitude toward vaccines among the young parent demographic, and, if necessary, to try to improve these perceptions. We found that a comparatively high population of parents did not have a good impression of vaccination and did not understand the purpose of vaccines, although most parents still intended to provide routine vaccines for their children. Consistent with previous reports [6], [7], [8], 95.9 % of parents answered that they were planning to provide vaccines for their children; nonetheless, the proportion of parents who had a positive impression or who were confident of their vaccine knowledge was relatively low. Parents’ knowledge is good when the vaccine coverage is optimal and vice versa [28], [29], [30], [31]. With the increase in the parents’ perception or knowledge improved, the vaccination behavior became preferable [31], [32], [33], [34]. The discrepancy in decision-making and attitude seen in our study in Japan may be because parents vaguely felt that routine preventive care is the right choice and/or their obligation, rather than because they genuinely understand the specific adverse sequelae that they are preventing by adhering to the standard vaccine schedule. For example, parents may take the Hib vaccine not because it prevents life-threatening bacterial meningitis but rather because it is one of several vaccines that are supposed to be taken routinely. Addressing this gap and parents’ choice to vaccinate based on a firm belief and trust is necessary for better vaccination coverage. Otherwise, the vaccine coverage would possibly drop down to the current level of parents’ understanding.

As an illustrative example, we note that the Hib vaccine is comparatively new in Japan; routine inoculation was only initiated in 2013, and as previously reported [31], the rate of parents contacted through this questionnaire who answered that they understood the efficacy and risk associated with the Hib vaccine was lower than for the other routine vaccines (Table 5). However, parents felt their knowledge of the HPV vaccine was deficient relative to the Hib vaccine. Other reports showed that HPV vaccine acceptance tended to be lower than other childhood vaccines [35], [36], [37], which was consistent with the result of the present survey. L. Helmkanp et al. report that the lack of confidence in the HPV vaccine was related to vaccine hesitancy [36]. In such similar situations, because they did not have a firm reason to vaccinate, parents often decide not to give vaccines to their children in the event of damaging information on vaccines arises, even though they were essential for their children’s health. Also, it can be assumed from the cases of the mumps vaccine or the HPV vaccine that parents will refuse vaccination although the adverse event in question is not accurate or relevant to the vaccine in question, or even though these vaccines are much more beneficial than harmful [9], [10], [24], [38]. Even when the vaccination coverage is seemingly high, studies indicate that continuous provision of information about vaccination is vital for parents to get their children vaccinated with confidence and maintain or improve the vaccination rate, more so for the HPV vaccine [36].

**Table 5:** Different understanding of each vaccine.

	Value	Std.Error	*t*-value	*p*-value
Hib (reference)	1.483	0.03	48.000	<0.001
PCV	−0.046	0.013	−3.611	<0.001
HB	−0.073	0.015	−4.975	<0.001
DPT-IPV	0.003	0.013	0.234	0.815
BCG	−0.066	0.017	−3.991	<0.001
MR	−0.100	0.022	−4.530	<0.001
Varicella	−0.107	0.024	−4.387	<0.001
HPV	0.581	0.038	15.331	<0.001

**Abbreviations:** Hib, *Haemophilus influenzae* type b; PCV, pneumococcal conjugate vaccine; HBV, hepatitis b virus; DPT-IPV, diphtheria/pertussis/tetanus/inactivated polio vaccine; BCG, Bacille de Calmette et Guérin; MR, measles/rubella; HPV, human papillomavirus.

According to our investigation, people received information about vaccines mainly from official institutions (doctors, notifications from the government, and the Maternal and Child Health Handbook [39]), and the information contained therein could thus improve the general impressions of vaccines. Previous reports state that information sources of vaccines are mainly physicians, pediatricians, and scientific information, and vaccine hesitancy is related to other information channels such as the Internet or social media [29], [40], [41], [42], [43], [44]. Furthermore, trust in doctors and good communication with physicians can reduce vaccine hesitancy [41], [45], [46], [47], [48]. Government officials and physicians (especially pediatricians) can work towards improving vaccine education and overall impression through these official channels. As first-time parents tend to have a good impression of vaccines when they feel they have a good understanding of the vaccine in question, according to our study, access to the correct information at an early stage is more likely effective than providing what essentially is remedial information later on. Additionally, people are more likely to form a negative impression about vaccines when they obtain information from their neighbors rather than through official channels. If proper knowledge is established among parents and their respective families, the target demographic will be more likely to introduce this information and/or provide access to the proper information sources to their close contacts, leading to the widespread of correct information rather than the viral spread of misinformation [41], [47], [49].

Next, we examined parents' attitudes toward the HPV vaccine specifically, because the inoculation rate in Japan is abysmally low regardless of the vaccine’s efficacy. A total of 21.2 % of parents in this survey population answered that they were planning to vaccinate their children, which is much a greater proportion of the population than the actual inoculation rate nationwide. Optimistically, we may consider these statistics as showing trends and tendencies toward recovering immunization coverage. However, as the responders within the current study are members of a maternity support site, this result is almost certainly an overestimation of the real population-level statistics because the survey population comprised parents who visited Ninps Lab that intended to provide scientifically plausible knowledge under the supervision of medical doctors; thus, they were already more likely to have a positive attitude toward vaccination. Nevertheless, there was still a fear of vaccination and a lack of understanding regarding vaccination among this survey population.

A total of 25.7 % of questionnaire respondents answered that they were in favor of the HPV vaccine, representing a far more negative impression relative to other vaccines, which is the case in other countries [35], [36], [37]. As in the previous report [50], [51], positive vaccine impressions significantly affected parents’ decision to provide vaccines for their infants in our study (*p* < 0.001). However, for the HPV vaccine, parents felt that they had understood the information about this vaccine comprehensively and still had a negative attitude toward vaccination against HPV based on the results of this survey. This may be because, when parents learn about the HPV vaccine, they gain information on potential severe adverse side effects. Additionally, parents tend to be susceptible to misinformation as they are more concerned and not confident about the HPV vaccine, and some seek the information about the vaccine from knowledge sources other than doctors or official sources [35], [36], [37]. Previous reports have demonstrated that negative and sensationalized broadcasting of events ostensibly occurring after vaccination is not relevant to actual vaccination uptake seen on the Internet or mass media reports [9], [24], [52]. However, when parents research the risks themselves, they tend to be somewhat biased by the exaggerated or misleading reporting of these events in the mass media and on social media and might ultimately misconstrue the reported events as a direct result of vaccination [53]. It is hence urgently necessary to properly inform and educate parents on choosing information sources correctly, particularly about their children’s health and wellbeing. To resolve the current situation, the provision of correct and adequate information by physicians and government officials, who are the main information sources for parents is important. These channels positively affect impressions of the HPV vaccine and are effective for improving parents’ knowledge of the HPV vaccine and the vaccine uptake, as evidenced in other previous reports as well [54], [55], [56], [57].

Additionally, the mediation analysis reported herein implies that a good impression of routine vaccination has a positive effect on impressions regarding the HPV vaccine specifically, thereby leading parents to ultimately decide to provide this vaccine to their children. This is probably because parents with a positive impression about vaccines tend to acquire information from reliable sources, and they trust and obtain vaccines [58]. Furthermore, they may acquire information about the HPV vaccine from the same reliable information sources. As M. Grandahl et al. implied, we conclude that a better understanding and impression of routine vaccination programs and other vaccines would positively affect the recovery of HPV vaccine coverage according to our analysis. To improve overall public health, specifically, that of the children, researchers and physicians are key players who have to provide more accurate, convincing, and comprehensive evidence about the HPV vaccine [50], [51], [59], and we should also strive to improve education on other vaccines within the general population.

There are some limitations to our study. First, the questionnaire was not organized based on a preliminary study. We did not adjust the questions for the study population and did not project the minimum required sample size to identify significant outcomes in our analysis. Thus, some finding could not be identified. Recall bias may have affected the results. The hesitant parents possibly remembered the information sources other than official sources that were supposed to be the ordinary information channels. However, the sample size was relatively large, and the results should be reliable to some extent. The pattern of the information source was similar to that of previous reports. Moreover, we should consider the lack of representativeness of the participants in Japan. Our survey population could be biased because the respondents voluntarily participated in the questionnaire surveys, and we did not actively recruit them. They were more likely to have a more positive attitude toward vaccination as compared with a more representative source population (i.e., all parents in Japan). The ratio of participants enrolled in our study who planned to provide their children with the HPV vaccine was much higher than the actual vaccination rate nationwide. However, the distribution of the places where participants were from was similar to that of the actual population, which might show some representativeness of our cohort. For future research efforts, we recommend constructing questions beforehand that would better evade standard biases and/or lead to acquiring more valuable data. For example, we may ask questions about specific problems that affect parents’ vaccine acceptance, such as side effects or access to care. Further, specific knowledge requirements of the parents should be identified, which can be useful in guiding interventions to improve vaccine knowledge and perceptions of parents. In any case, our findings suggest that the provision of more comprehensive and more effectively delivered information to parents is necessary to improve vaccine coverage and the overall state of public health.

## Conclusions

In this study, we examined parents’ attitudes toward vaccination through a comprehensive and large-scale questionnaire survey. We conclude that we need to provide parents with adequate information about vaccines through various official channels to cultivate a better attitude toward vaccination, thereby ultimately improving the currently low HPV vaccine coverage in Japan. Our findings may guide future research efforts and directly inform health policy, health education programming, and medical decision-making.

## Funding

This research did not receive any specific grant from funding agencies in the public, commercial, or not-for-profit sectors.

## Data Availability

The data used for this study, although unavailable in a public repository, will be made available upon reasonable request to the corresponding author.

## Declaration of Competing Interest

The authors declare that they have no known competing financial interests or personal relationships that could have appeared to influence the work reported in this paper.

## Data Availability

Data will be made available on request.

## References

[1] Pollard A.J., Bijker E.M. (2021). A guide to vaccinology: from basic principles to new developments. Nat Rev Immunol.

[2] Fogleman C., Leaman L. (2019). Prophylactic vaccination against human papillomavirus to prevent cervical cancer and its precursors. Am Fam Phys.

[3] Guo F., Cofie L.E., Berenson A.B. (2018). Cervical cancer incidence in Young, U.S. Females after human papillomavirus vaccine Introduction. Am J Prev Med.

[4] Harper D.M., DeMars L.R. (2017). HPV vaccines – A review of the first decade. Gynecol Oncol.

[5] Palmer T., Wallace L., Pollock K.G., Cuschieri K., Robertson C., Kavanagh K. (2019). Prevalence of cervical disease at age 20 after immunisation with bivalent HPV vaccine at age 12–13 in Scotland: Retrospective population study. BMJ.

[6] World Health Organization. WHO vaccine-preventable diseases: Monitoring system. 2020 Global Summary 2020. https://apps.who.int/immunization_monitoring/globalsummary/. [accessed 15 July 2020].

[7] WHO. Timor-Leste : WHO and UNICEF estimates of immunization coverage : 2019 2020:1–17.

[8] Ministry of Health Labour and Welfare (Japan). https://www.mhlw.go.jp/topics/bcg/other/5.html [accessed 12 April 2022].

[9] Ikeda S., Ueda Y., Yagi A., Matsuzaki S., Kobayashi E., Kimura T. (2019). HPV vaccination in Japan: What is happening in Japan?. Expert Rev Vaccines.

[10] Simms K.T., Hanley S.J.B., Smith M.A., Keane A., Canfell K. (2020). Impact of HPV vaccine hesitancy on cervical cancer in Japan: A modelling study. Lancet Public Heal.

[11] Sadaf A., Richards J.L., Glanz J., Salmon D.A., Omer S.B. (2013). A systematic review of interventions for reducing parental vaccine refusal and vaccine hesitancy. Vaccine.

[12] Who (2014). Report of the Sage Working Group on. WHO COVID-19 Glob Data.

[13] Salmon D.A., Dudley M.Z., Glanz J.M., Omer S.B. (2015). Vaccine Hesitancy: Causes, Consequences, and a Call to Action. Am J Prev Med.

[14] Dubé È., Farrands A., Lemaitre T., Boulianne N., Sauvageau C., Boucher F.D. (2019). Overview of knowledge, attitudes, beliefs, vaccine hesitancy and vaccine acceptance among mothers of infants in Quebec. Canada Hum Vaccin Immunother.

[15] Burki T. (2019). Vaccine misinformation and social media. Lancet Digit Heal.

[16] Ozawa S., Stack M.L. (2013). Public trust and vaccine acceptance-international perspectives. Hum Vaccines Immunother.

[17] Siciliani L., Wild C., McKee M., Kringos D., Barry M.M., Barros P.P. (2020). Strengthening vaccination programmes and health systems in the European Union: A framework for action. Health Policy (New York).

[18] Larson H.J., Sahinovic I., Balakrishnan M.R., Simas C. (2021). Vaccine safety in the next decade: why we need new modes of trust building. BMJ Glob Heal.

[19] Ampofo A.G., Boyes A.W., Khumalo P.G., Mackenzie L. (2022). Improving knowledge, attitudes, and uptake of cervical cancer prevention among female students: A systematic review and meta-analysis of school-based health education. Gynecol Oncol.

[20] Nehal K.R., Steendam L.M., Ponce M.C., van der Hoeven M., Smit G.S.A. (2021). Worldwide vaccination willingness for covid-19: A systematic review and meta-analysis. Vaccines.

[21] Cooper S., Schmidt B.-M., Sambala E.Z., Swartz A., Colvin C.J., Leon N. (2021). Factors that influence parents’ and informal caregivers’ views and practices regarding routine childhood vaccination: a qualitative evidence synthesis. Cochrane Database Syst Rev.

[22] Della Polla G., Pelullo C.P., Napolitano F., Angelillo I.F. (2020). HPV vaccine hesitancy among parents in Italy: a cross-sectional study. Hum Vaccin Immunother.

[23] Bianco A., Mascaro V., Zucco R., Pavia M. (2019). Parent perspectives on childhood vaccination: How to deal with vaccine hesitancy and refusal?. Vaccine.

[24] Mizumachi K., Aoki H., Kitano T., Onishi T., Takeyama M., Shima M. (2021). How to recover lost vaccine acceptance? A multi-center survey on HPV vaccine acceptance in Japan. J Infect Chemother.

[25] Yagi A., Ueda Y., Egawa-Takata T., Tanaka Y., Morimoto A., Terai Y. (2016). Development of an efficient strategy to improve HPV immunization coverage in Japan. BMC Public Health.

[26] Fujiwara H., Takei Y., Ishikawa Y., Saga Y., Machida S., Taneichi A. (2013). Community-based interventions to improve HPV vaccination coverage among 13- to 15-year-old females: measures implemented by local governments in Japan. PLoS One.

[27] Imai K., Keele L., Tingley D., Yamamoto T. (2011). Unpacking the black box of causality: Learning about causal mechanisms from experimental and observational studies. Am Polit Sci Rev.

[28] Alshammari T.M., Subaiea G.M., Hussain T., Moin A., Yusuff K.B. (2018). Parental perceptions, attitudes and acceptance of childhood immunization in Saudi Arabia: A cross sectional study. Vaccine.

[29] Alawneh I., Saymeh A., Yasin A., Alawneh M., Al-Tatari H. (2020). Vaccines attitudes, concerns, and information sources reported by parents of young children among North Palestinian parents. Adv Prev Med.

[30] Sabahelzain M.M., Moukhyer M., Bosma H., van den Borne B. (2022). Determinants of measles vaccine hesitancy among sudanese parents in khartoum state, sudan: A cross-sectional study. Vaccines.

[31] Saitoh A., Saitoh A., Katsuta T., Mine M., Kamiya H., Miyairi I. (2020). Effect of a vaccine information statement (VIS) on immunization status and parental knowledge, attitudes, and beliefs regarding infant immunization in Japan. Vaccine.

[32] Basheer S.A., Kumar R., Viwattanakulvanid P., Yaha M.B., Somrongthong R. (2021). Effect of interpersonal communication training program on child’s immunization among mothers living in kebbi state of Nigeria. J Ayub Med Coll Abbottabad.

[33] Lv K., Zhao J., Zhang P. (2021). The effect of community comprehensive nursing using scenario-based health education on the infant and young child immunization rates. Am J Transl Res.

[34] Tkaczyszyn K., Kuchar E., Augustynowicz E., Szenborn L. (2021). The impact of a single educational lecture on the vaccine confidence among pregnant women and young mothers. Vaccines.

[35] Furgurson K.F., Sandberg J.C., Hsu F.C., Mora D.C., Quandt S.A., Arcury T.A. (2019). HPV knowledge and vaccine uptake among Mexican-born farmworkers in North Carolina. Health Promot Pract.

[36] Helmkamp L.J., Szilagyi P.G., Zimet G., Saville A.W., Gurfinkel D., Albertin C. (2021). A validated modification of the vaccine hesitancy scale for childhood, influenza and HPV vaccines. Vaccine.

[37] Ogilvie G., Anderson M., Marra F., McNeil S., Pielak K., Dawar M. (2010). A population-based evaluation of a publicly funded, school-based HPV vaccine program in British Columbia, Canada: Parental factors associated with HPV vaccine receipt. PLoS Med.

[38] Kitano T., Nishikawa H., Onaka M., Ishihara M., Nishiyama A., Yoshida S. (2018). Questionnaire survey on mumps vaccination for parents in Nara Prefecture. Japan Pediatr Int.

[39] Ministry of Health Labour and Welfare. Maternal and Child Health Handbook 2022. https://www.mhlw.go.jp/content/11900000/000765705.pdf [accessed 24 March 2022].

[40] Tabacchi G., Costantino C., Cracchiolo M., Ferro A., Marchese V., Napoli G. (2017). Information sources and knowledge on vaccination in a population from southern Italy: The ESCULAPIO project. Hum Vaccines Immunother.

[41] Wheeler M., Buttenheim A.M. (2013). Parental vaccine concerns, information source, and choice of alternative immunization schedules. Hum Vaccines Immunother.

[42] Yörük S., Güler D. (2021). Factors associated with pediatric vaccine hesitancy of parents: a cross-sectional study in Turkey. Hum Vaccines Immunother.

[43] Gkentzi D., Tsagri C., Kostopoulou E., Fouzas S., Vantarakis A., Dimitriou G. (2021). Attitudes and beliefs of parents about routine childhood vaccination in Greece. Hum Vaccin Immunother.

[44] Holroyd T.A., Howa A.C., Delamater P.L., Klein N.P., Buttenheim A.M., Limaye R.J. (2021). Parental vaccine attitudes, beliefs, and practices: initial evidence in California after a vaccine policy change. Hum Vaccines Immunother.

[45] Sabahelzain MM, Moukhyer M, Bosma H, van den Borne B. Determinants of measles vaccine hesitancy among Sudanese parents in Khartoum State, Sudan: a Cross-Sectional Study. Vaccines 2021;10. 10.3390/vaccines10010006. 10.3390/vaccines10010006 PMC8780692 35062667

[46] Giannakou K., Kyprianidou M., Hadjikou A., Fakonti G., Photiou G., Tzira E. (2021). Knowledge of mothers regarding children’s vaccinations in Greece: an online cross-sectional study. BMC Public Health.

[47] Mayerová D., Abbas K. (2021). Childhood immunisation timeliness and vaccine confidence by health information source, maternal, socioeconomic, and geographic characteristics in Albania. BMC Public Health.

[48] Nowak G.J., Cacciatore M.A. (2017). Parents’ confidence in recommended childhood vaccinations: Extending the assessment, expanding the context. Hum Vaccines Immunother.

[49] Dudley M.Z., Limaye R.J., Omer S.B., O’Leary S.T., Ellingson M.K., Spina C.I. (2020). Factors associated with referring close contacts to an app with individually tailored vaccine information. Vaccine.

[50] Cocchio S., Bertoncello C., Baldovin T., Fonzo M., Bennici S.E., Buja A. (2020). Awareness of HPV and drivers of HPV vaccine uptake among university students: A quantitative, cross-sectional study. Heal Soc Care Community.

[51] Roura-Monllor J., Nieves-Muñoz J., Ortiz A.P., Romaguera J. (2018). HPV knowledge, vaccine knowledge, and vaccine acceptance in women with cervical cytology anomalies attending colposcopy clinics in Puerto Rico. Int J Gynecol Obstet.

[52] Suzuki S., Hosono A. (2018). No association between HPV vaccine and reported post-vaccination symptoms in Japanese young women: Results of the Nagoya study. Papillomavirus Res.

[53] Beavis A., Krakow M., Levinson K., Rositch A.F. (2018). Reasons for Lack of HPV vaccine initiation in NIS-Teen over time: Shifting the focus from gender and sexuality to necessity and safety. J Adolesc Heal.

[54] Bianco A., Pileggi C., Iozzo F., Nobile C.G.A., Pavia M. (2014). Vaccination against human papilloma virus infection in male adolescents: Knowledge, attitudes, and acceptability among parents in Italy. Hum Vaccines Immunother.

[55] Navarro-Illana P., Navarro-Illana E., Vila-Candel R., Díez-Domingo J. (2018). Drivers for human papillomavirus vaccination in Valencia (Spain). Gac Sanit.

[56] Newman P.A., Logie C.H., Lacombe-Duncan A., Baiden P., Tepjan S., Rubincam C. (2018). Parents’ uptake of human papillomavirus vaccines for their children: A systematic review and meta-analysis of observational studies. BMJ Open.

[57] Naoum P., Athanasakis K., Zavras D., Kyriopoulos J., Pavi E. (2022). Knowledge, perceptions and attitudes toward HPV vaccination: a survey on parents of girls aged 11–18 years old in Greece. Front Glob Women’s Heal.

[58] Kessels S.J.M., Marshall H.S., Watson M., Braunack-Mayer A.J., Reuzel R., Tooher R.L. (2012). Factors associated with HPV vaccine uptake in teenage girls: A systematic review. Vaccine.

[59] Napolitano F., Navaro M., Vezzosi L., Santagati G., Angelillo I.F. (2018). Primary care pediatricians’ attitudes and practice towards hpv vaccination: A nationwide survey in Italy. PLoS One.

